# Community Health and Demographic Surveillance System for Noncommunicable Disease Epidemiology Among Adults in Rural and Urban Zimbabwe, 2024-2029: Protocol for a Longitudinal Surveillance Study

**DOI:** 10.2196/89292

**Published:** 2026-07-10

**Authors:** Richard Makurumidze, Terrence Rudado Musekiwa, Justice Mudavanhu, Newten Handireketi, Manes Munyanyi, Cremance Tshuma, Marvellous Mhloyi, Justen Manasa, Jonathan Arthur Matenga, Simbarashe Rusakaniko

**Affiliations:** 1 Faculty of Medicine and Health Sciences, University of Zimbabwe, Parirenyatwa Group of Hospitals Complex, Mazowe Street, Harare, Zimbabwe, 263 772759296; 2 Ministry of Health and Child Welfare, Harare, Zimbabwe; 3 Department of Health Sciences, Bindura University of Science Education, Bindura, Zimbabwe; 4 Faculty of Social and Behavioural Sciences, University of Zimbabwe, Harare, Zimbabwe

**Keywords:** health and demographic surveillance system, noncommunicable diseases, epidemiology, rural-urban, Zimbabwe

## Abstract

**Background:**

Zimbabwe currently faces a rapidly escalating burden of noncommunicable diseases (NCDs) concurrently with persistent communicable disease challenges, resulting in profound epidemiological differences between rural and urban populations. To effectively address this evolving epidemiological landscape and guide evidence-based public health interventions, reliable and high-quality longitudinal data are essential for capturing temporal shifts and contextual determinants often overlooked by conventional health information systems.

**Objective:**

This protocol details the methodology for establishing a health and demographic surveillance system (HDSS), a longitudinal, population-based cohort designed to continuously monitor the prevalence, incidence, and key determinants of NCDs, specifically cardiovascular diseases and diabetes, and their associated risk factors in selected rural (Mt Darwin) and urban (Bindura) sites in Mashonaland Central Province over a 5-year period (2024‐2029).

**Methods:**

The HDSS uses a stratified multistage sampling design to recruit adults (aged ≥18 y) residing across 2 rural and 2 urban wards. Initial activities include comprehensive community profiling and household mapping, followed by a rigorous baseline survey, with systematic follow-ups scheduled every year. Data encompass essential domains such as vital events, migration patterns, detailed social determinants of health, health behaviors, self-reported and clinically assessed NCDs, physical examinations (including height, weight, blood pressure, and waist/hip circumference), biochemical markers (fasting glucose, lipid profiles, and urine sodium/creatinine), and standardized verbal autopsies. Data capture will make use of REDCap (Research Electronic Data Capture) to facilitate real-time data entry and validation. The protocol is underpinned by rigorous quality assurance procedures, continuous community engagement, and comprehensive ethical oversight.

**Results:**

The Zimbabwe HDSS received ethical approval on July 30, 2024 (MRCZ/A/3191). Baseline data collection was completed in Mt Darwin (rural site) in December 2024 and in Bindura (urban site) in March 2025. Statistical analysis of baseline NCD prevalence, risk factor distributions, and rural-urban comparisons is underway, with findings expected to be submitted for publication by the end of 2026. The first annual longitudinal follow-up round is planned for the second quarter of 2027, subject to funding availability, with subsequent rounds scheduled annually through 2029. Longitudinal incidence estimates and NCD trend analyses are expected to be published progressively from 2026 to 2029.

**Conclusions:**

This community HDSS addresses a critical evidence deficit within Zimbabwe’s national health information infrastructure. By implementing an ethical, sustainable, and community-engaged research methodology, the HDSS serves as a potent regional model. It is designed to generate actionable, policy-relevant data for national health authorities and stakeholders, thereby accelerating the country’s NCD prevention and control agenda while simultaneously acting as an enduring platform for academic innovation, capacity building, and policy translation efforts targeting both rural and urban health disparities.

## Introduction

### Burden of Noncommunicable Diseases

Noncommunicable diseases (NCDs)—cardiovascular diseases, diabetes, cancer, and chronic respiratory diseases—account for 71% of global deaths annually, with low- and middle-income countries bearing a disproportionate 80% of NCD mortality [1,2]. Sub-Saharan Africa faces a particularly complex dual burden: persistently high communicable disease rates coexist with rapidly rising NCD prevalence, creating unprecedented challenges for health systems designed primarily for acute infectious diseases [3]. Zimbabwe exemplifies this epidemiological transition, with NCDs now accounting for 32% of all deaths in 2023, hypertension prevalence ranging from 18% to 38% across different populations, and diabetes increasing dramatically from 0.44% in the pre-1980 period to 5.7% in recent years [4,5]. Critically, many cases remain undiagnosed and untreated, with individuals accessing care only when serious complications arise [6].

Rural-urban disparities add substantial complexity to this landscape. Urban residents face heightened exposure to NCD risk factors, including energy-dense diets, physical inactivity, tobacco use, and alcohol consumption [7], while rural populations encounter limited health infrastructure, severe personnel shortages, inadequate diagnostic capacity, and substantial treatment delays [8]. Migration and urbanization fundamentally alter disease risk profiles as individuals transition between environments [9]. This geographic divide is further complicated by the NCD-HIV intersection, where poor chronic disease control has emerged as the strongest predictor of mortality in the antiretroviral therapy era [10].

### Gaps in Existing Health Information Systems

Current surveillance systems suffer critical inadequacies that impede effective NCD response. Weak Civil Registration and Vital Statistics systems create fundamental gaps in understanding mortality dynamics, with many deaths occurring outside facilities and never recorded [11,12]. Facility-based surveillance captures only individuals accessing formal health care, systematically missing those who are undiagnosed, use traditional providers, or face access barriers [13]. This dramatically underestimates burden for NCDs with substantial asymptomatic populations [14]. Cross-sectional surveys cannot track longitudinal trends, assess incidence, or establish temporal relationships [15]. Self-reported data substantially underestimate NCD prevalence compared to biomarker measurement [14]. Systems lack the geographic granularity needed for targeted interventions [16].

### Limitations of Demographic and Health Surveys

Zimbabwe has a well-established tradition of population-based health surveys, anchored by the Demographic and Health Survey (DHS) program, which has been conducted 7 times since 1988. DHSs, while valuable, have significant constraints for NCD surveillance. Episodic conduct at typically 5-year intervals creates temporal gaps during which critical changes occur undetected [16]. The cross-sectional design precludes tracking individuals, assessing incidence, or establishing causal relationships [17], particularly problematic for NCDs developing over decades [10]. DHS has historically focused on maternal-child health, with limited adult NCD coverage [18]. Rapid sociocultural changes necessitate continuous surveillance to capture evolving risk profiles [19].

### Health and Demographic Surveillance Systems: A Solution

Health and demographic surveillance systems (HDSSs) provide continuous, longitudinal, population-based measurement of demographic, health, and social indicators within geographically defined populations [20,21]. HDSSs conduct regular household visits at intervals of 4 to 12 months, systematically updating births, deaths, migrations, and health events for all residents, enabling accurate calculation of rates and individual-level tracking over time [22]. This longitudinal design allows incidence assessment, prospective risk factor identification, intervention evaluation, and temporal trend monitoring that are impossible with cross-sectional surveys [15].

HDSSs differ fundamentally from other surveillance approaches in ways directly relevant to NCD monitoring. Unlike facility-based surveillance that captures only health care–seeking individuals, HDSSs conduct active community-based surveillance reaching all residents regardless of care-seeking behavior, health status, or socioeconomic position [11]. Unlike sentinel systems that monitor selected populations or facilities, HDSSs provide comprehensive data for entire defined populations, enabling unbiased prevalence and incidence estimation [23]. Unlike DHS, which provides periodic snapshots, HDSSs generate continuous real-time data enabling detection of emerging trends and rapid public health response [24].

Concrete examples demonstrate HDSS value for NCD surveillance. Verbal autopsies conducted in 6 eastern and southern African HDSSs revealed stable NCD mortality rates from 1995 to 2019 despite substantial declines in communicable disease mortality—epidemiological patterns that would be invisible to episodic cross-sectional surveys [25]. In rural South Africa, HDSS data showed that life-years lost to NCDs exceeded pre-HIV epidemic levels by 2014‐2018, demonstrating the system’s capacity to detect critical epidemiological transitions and inform policy priorities [19].

HDSSs employ standardized instruments ensuring data comparability across sites and over time [21], maintain population-based sampling frames covering all residents [20], and integrate multiple data sources, including household surveys, health facility records, and vital events registration [26]. They serve as platforms for nested studies, randomized trials, and intervention evaluations [27], generating evidence directly applicable to policy and practice.

### Rationale for Rural-Urban HDSS in Zimbabwe

Establishing an HDSS spanning rural and urban Zimbabwe strategically addresses identified surveillance gaps through direct rural-urban comparison. The rationale is 3-fold. First, NCD burden, determinants, and health consequences differ substantially between rural and urban settings, requiring context-specific evidence [17]. Second, Zimbabwe’s rapid urbanization, with increasing rural-to-urban migration, fundamentally alters population health profiles, necessitating surveillance systems that capture these dynamic transitions [28]. Third, health system capacity, infrastructure, service delivery models, and resource availability differ markedly between rural and urban areas [29], requiring geographically tailored intervention strategies.

### Study Aims

This paper describes the establishment of a Zimbabwe HDSS to address critical NCD surveillance gaps through 4 specific aims:

1. Establish a longitudinal, population-based HDSS spanning rural and urban sites to generate accurate, timely data on demographic dynamics, NCD burden, risk factors, and health care access through continuous household surveillance with regular follow-up.
2. Characterize prevalence, incidence, and temporal trends of major NCDs—hypertension, diabetes, cardiovascular diseases, and chronic respiratory diseases—with particular emphasis on identifying undiagnosed and untreated cases through active case-finding and objective biomarker measurement.
3. Quantify rural-urban differences in NCD burden, risk factor exposure profiles, health care–seeking behavior, treatment access, and health outcomes to inform geographically targeted interventions and evidence-based resource allocation.
4. Generate actionable evidence informing national NCD policy development, strategic planning, resource allocation decisions, and intervention design through systematic integration with existing health systems and continuous stakeholder engagement.

By addressing fundamental gaps in existing surveillance infrastructure, this HDSS will provide the continuous, geographically granular, longitudinal data essential for evidence-based NCD prevention and control in Zimbabwe’s rapidly evolving epidemiological landscape.

## Methods

### Study Design

**Figure 1.**
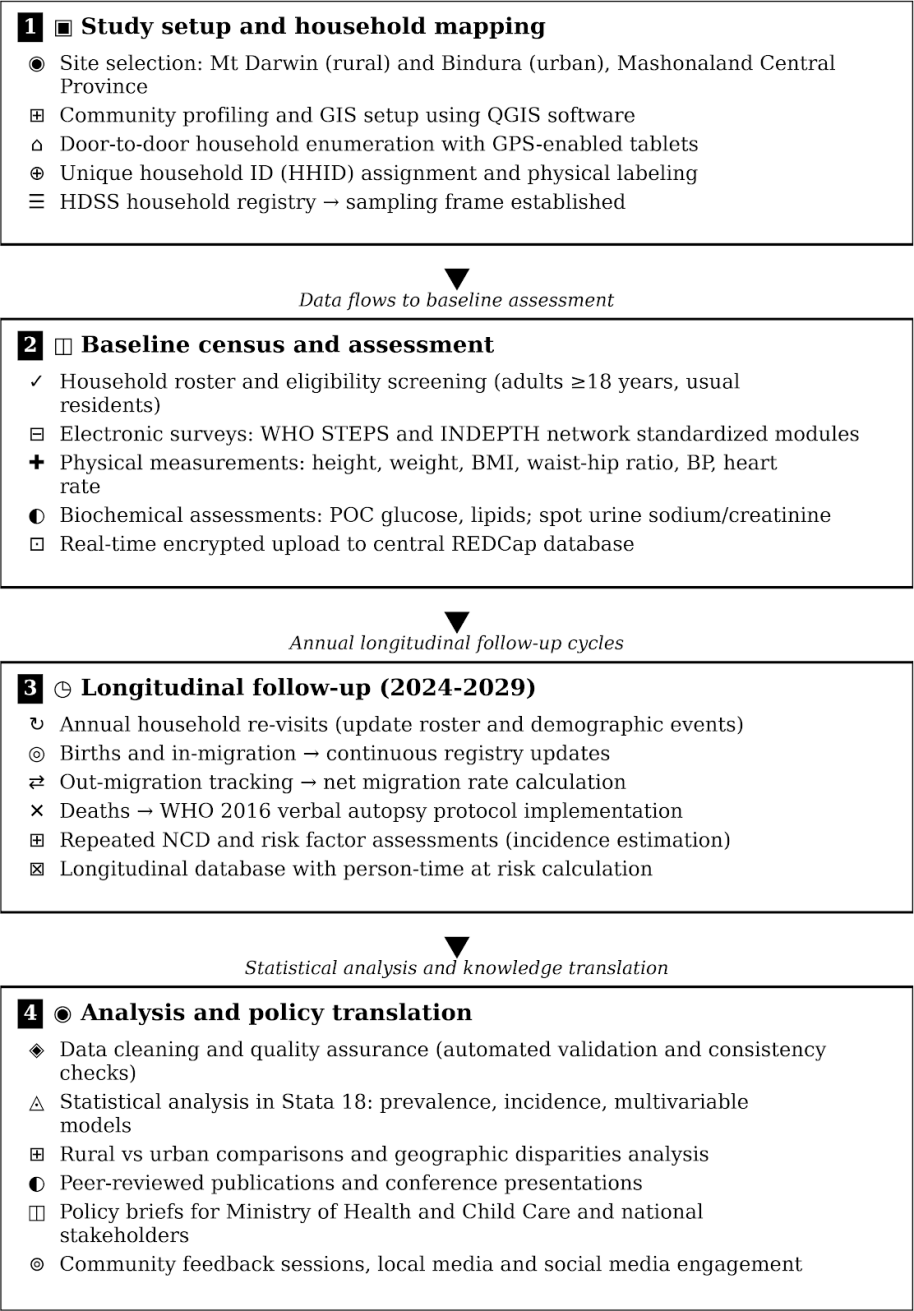
Health and demographic surveillance system workflow in Mashonaland Central Province, Zimbabwe, 2025. BP: blood pressure; GIS: geographic information system; HDSS: health and demographic surveillance system; NCD: noncommunicable disease; POC: point-of-care; REDCap: Research Electronic Data Capture; WHO STEPS: World Health Organization STEPwise approach to NCD risk factor surveillance.

This protocol outlines the methodology for a prospective, longitudinal HDSS established in geographically defined rural and urban communities in Zimbabwe. The HDSS is designed to collect, monitor, and compare comprehensive health, demographic, and behavioral data critical to NCDs over a continuous 5-year period (2024‐2029). This systematic approach aligns with established HDSS methodologies that enable dynamic, population-based measurement and repeated assessments, which are vital for characterizing the evolution of NCD burdens across distinct social and economic contexts. A schematic flowchart providing an overview of the HDSS workflow across all 4 phases has been developed (Figure 1).

### Study Setting

The HDSS implementation will occur across 2 purposively selected districts within Mashonaland Central Province, Zimbabwe: Mt Darwin (rural site) and Bindura (urban site). Mt Darwin District encompasses approximately 1500 km^2^, hosting an estimated population of around 120,000 residents. The population predominantly relies on subsistence and small-scale commercial farming (eg, maize and tobacco), with sociodemographic indicators suggesting moderate educational attainment and agriculture-based household income. Health care services are provided by the Mt Darwin District Hospital and smaller rural clinics. Bindura District represents the urban setting, covering roughly 2800 km^2^ with an estimated population of 140,000 distributed across 14 wards. Bindura exhibits higher population density and a diversified economy, including mining (Bindura Nickel Corporation), trading, and academic communities linked to Bindura University. Health infrastructure is comparatively advanced, featuring the Bindura Provincial Hospital (a 200-bed referral facility) complemented by an extended clinic network. The HDSS will utilize 4 purposively selected wards (2 rural and 2 urban) to capture these socioeconomic and epidemiological contrasts.

### Community Profiling and Household Mapping

The initial phase involves rigorous community profiling, which synthesizes national census data, local administrative knowledge, geographic information systems (GIS), and satellite imagery analysis. This process systematically documents contextual factors, including local history; population characteristics (demographic and socioeconomic indicators); transportation and communication systems; employment and income sources; housing patterns; educational facilities (including information and communication technology use and teacher capacity); health facilities; financial institutions; nongovernmental organizations; recreational centers; agricultural and veterinary services; places of worship; and environmental degradation (eg, deforestation for tobacco curing, artisanal mining, and silted reservoirs). Governmental schemes, including state-administered agricultural support, social cash transfers, food assistance, and public works programs operating in the ward, were assessed. As part of community profiling, the availability of mobile network providers within each ward is documented as a proxy indicator of network coverage and field communication feasibility. It is acknowledged that enumeration of service providers is a limited proxy that does not capture household- or individual-level digital access, affordability, internet literacy, or device ownership; comprehensive digital readiness assessment is beyond the scope of the current protocol but is identified as an important priority for future HDSS rounds.

Household mapping is conducted as a structured, multistep process to generate a complete and reproducible sampling frame for longitudinal surveillance. First, existing Enumeration Areas (EAs) from the most recent national census are obtained from the Zimbabwe National Statistics Agency and imported into a GIS platform (QGIS) to delineate EA boundaries for the selected wards. Second, trained field mappers conduct a door-to-door household listing within each EA using handheld GPS-enabled tablets preloaded with custom forms developed in REDCap (Research Electronic Data Capture), recording the precise geographic coordinates of each dwelling, its EA code, and basic dwelling characteristics (eg, structure type and occupancy status). Third, each residential structure that meets the HDSS definition of a household (people who “eat from the same pot” and usually sleep in the dwelling for at least 3 of the last 6 mo) is assigned a unique household identification (HHID) code, which is physically labeled on the dwelling (eg, paint or sticker) and simultaneously captured electronically. Fourth, the georeferenced household list (HHID, EA code, and GPS coordinates) is exported from the data collection platform and integrated into the central HDSS database, where it is linked to the GIS layer to enable spatial checks, duplicate detection, and verification of coverage. The resulting quality-checked household register constitutes the sampling frame for all baseline and follow-up HDSS rounds.

### Study Population and Sampling

The study population includes all adults aged 18 years and older who are usual residents in the defined rural and urban HDSS areas (ie, the 4 selected wards) at the time of the baseline census. For operational purposes, a “usual resident” is defined as an individual who has lived in the household for at least 3 of the previous 6 months or who intends to stay for at least 6 months, consistent with HDSS standards. As an HDSS, the system aims for exhaustive inclusion of all eligible adults within the mapped households rather than a sample-based design.

Within each mapped household (HHID), fieldworkers use a standardized roster module on the tablet to list all household members, recording name, sex, age, relationship to head of household, and residency status. Adults (aged ≥18 y) who meet the residency criterion and provide informed consent are included and invited to complete individual-level interviews and measurements. Exclusion criteria are (1) individuals younger than 18 years; (2) visitors or short-term guests who do not meet the residency definition; (3) individuals who are unable to provide informed consent and for whom no appropriate consenting procedure is approved; and (4) individuals who explicitly decline participation. For temporarily absent eligible adults (eg, at work or school), at least 2 repeat visits are scheduled at different times and days before classifying them as nonresponders for that round. This approach ensures near-complete coverage of the adult resident population in both communities.

Within the 4 selected wards, all mapped households constitute the sampling frame, from which a systematic sample of households is drawn. Within each sampled household, all adults aged 18 years and older who meet the residency criterion are identified and invited to participate. Although the HDSS does not enroll every eligible adult resident because of resource constraints, this probability-based household sampling strategy, combined with formal power calculations, ensures that the resulting sample size is sufficient to address the primary study objectives. Using a baseline hypertension prevalence estimate of 30%, consistent with published data from Zimbabwe and comparable sub-Saharan African settings, a margin of error of ±2%, a 95% CI, and a design effect of 1.5 to account for household clustering, a minimum sample of approximately 3000 adults per site is required for robust prevalence estimation. This yields a total minimum analytical sample of 6000 adults across both rural and urban sites.

For the detection of a modest 2% absolute annual change in hypertension or diabetes prevalence (eg, from 30% to 32%) between surveillance rounds, with 80% statistical power and a 2-sided α of .05, a minimum of approximately 3500 participants per site per round is required. The estimated adult population across the 4 HDSS wards substantially exceeds this threshold, supporting the longitudinal objectives of the study. For rural-urban comparative analyses, equal allocation across the 2 sites provides 80% power to detect an absolute difference of 3% to 5% in key NCD outcomes. Subgroup analyses by sex, age group, and socioeconomic stratum are designated as exploratory, and appropriate corrections for multiple comparisons will be applied. Sample size calculations were performed in accordance with the WHO STEPwise approach to NCD risk factor surveillance (WHO STEPS) sample size guidance for population-based NCD surveys.

### Data Collection Methods

Baseline and subsequent follow-up rounds will be conducted via structured, interviewer-administered surveys using REDCap tablet-based electronic questionnaires. These instruments have been developed by adapting core modules from the WHO STEPS [30] and the standardized DHS tools of the INDEPTH Network [20], ensuring alignment with established population-based surveillance practice. Household-level forms are linked to each HHID and capture location geocoordinates (imported directly from the mapping module), asset ownership, housing conditions, water and sanitation status, and records of vital events (births, deaths, and in- and out-migrations) since the last round. Individual-level forms are linked via a unique individual identification (INDID) nested within each HHID and address core sociodemographic factors (age, sex, marital status, education level, and occupation), women’s empowerment, and exposure to gender-based violence. Health-related modules capture self-reported history of NCDs (hypertension, diabetes, and cardiovascular disease), mental health status, and key communicable diseases (HIV, tuberculosis, and malaria), as well as reproductive health metrics. For each reported condition, data are also collected on prior diagnosis by a health professional, current treatment status, and type of medication, enabling estimation of the diagnosis-treatment-control cascade for key conditions. Behavioral risk factors assessed include tobacco use, alcohol consumption, dietary patterns, physical activity, and sexual behavior, with skip patterns and range checks built into the electronic tools to minimize data entry errors. All items are drawn from, or adapted directly from, WHO STEPS and INDEPTH Network instruments that have been widely used and validated in demographic and health surveillance sites in sub-Saharan Africa, supporting their reliability and suitability for this target population.

The household questionnaire additionally captures individual-level items on mobile phone ownership (personal or shared), primary connectivity mode (SMS, mobile data, or Wi-Fi), and internet access, providing a baseline characterization of digital access at the household and individual levels to contextualize NCD communication and service delivery in both rural and urban settings.

### Physical and Biochemical Measurements

Standardized physical assessments will be performed by trained nurses or clinical research assistants in accordance with WHO STEPS field measurement protocols using calibrated equipment [30]. These measurements include height, weight, BMI, waist and hip circumference, blood pressure (BP), and heart rate. BP and heart rate are assessed using validated, automated digital sphygmomanometers, which eliminate observer bias and terminal digit preference associated with manual auscultation. Field staff will ensure that participants are seated quietly with their back supported, feet flat on the floor, and arm supported at heart level, using an appropriately sized cuff. Following an initial 5 minutes of seated rest, 3 sequential BP and heart rate readings will be taken at 3-minute intervals. This 5-minute interval strictly adheres to the standardized WHO STEPS protocol for population-based field surveys. Taking 3 measurements allows for the accommodation of transient anxiety or the alerting reaction; the first reading is discarded, and the mean of the second and third readings is used in analyses to provide a reliable estimate of resting BP. To eliminate transcription errors, all readings are documented directly into the REDCap electronic case report forms on the field tablets, which are programmed with automated biological range checks. Height and weight measurements are taken using a stadiometer and digital scale, respectively, calibrated daily, and recorded to the nearest 0.1 cm and 0.1 kg, with height measured in the Frankfort plane. Central obesity is defined by a waist-to-hip ratio exceeding 0.85 in women and 0.95 in men. Biochemical assessments require participants to undergo an 8- to 12-hour overnight fast; finger-stick capillary blood samples are analyzed using point-of-care devices (lipidometers and glucometers) to determine fasting blood glucose, total cholesterol, fasting triglycerides, and high-density lipoprotein. A random spot urine sample (20 mL) is collected for estimation of urinary sodium and creatinine, and, where consent is provided, venous blood samples are taken for further laboratory tests (eg, full blood count and liver function tests). All measurements are recorded directly into the electronic data collection tools and automatically linked to the participant’s INDID.

### Follow-Up Procedures

Following the baseline census, longitudinal data collection rounds will be implemented annually over the 5-year surveillance period. Using the existing household and individual registers, field teams revisit each HHID and reverify the household roster, updating entries for births, deaths, in-migrations, out-migrations, and changes in marital status. Migration events are classified and dated using standard HDSS definitions, enabling calculation of person-time at risk and net migration rates. Migration events are identified and documented during each annual household roster update. New residents found in a previously mapped household who meet the standard residency criterion (at least 3 of the last 6 mo or intending to stay at least 6 mo) are classified as in-migrants, assigned a unique individual ID, and enrolled prospectively, with their origin and date of arrival recorded. Previously enrolled individuals confirmed by household members to have permanently departed the HDSS ward boundaries are classified as out-migrants, with their date of departure, destination, and reason for migration recorded and their follow-up record closed from that date, contributing person-time up to departure. Individuals who return after out-migration are reenrolled under their original ID, ensuring longitudinal continuity.

At each round, eligible adults are recontacted for updated interviews and measurements to estimate NCD prevalence and incidence, monitor changes in health-related behaviors and attitudes, and track risk factors over time. All recorded deaths are subjected to verbal autopsy using the WHO 2016 [31] standardized instrument administered on tablets; data are processed with automated cause-of-death assignment tools (eg, InterVA or InSilicoVA), given the limitations of vital registration in this setting.

Participant retention is managed through a structured recontact protocol whereby temporarily absent individuals receive at least 2 repeat visits at different times before being recorded as noncontactable for that round. At each round, the baseline characteristics of participants lost to follow-up will be compared with those retained to assess and account for potential attrition bias.

### Data Management and Quality Assurance

A comprehensive data management framework underpins the HDSS. Data capture uses an electronic system built on REDCap with a longitudinal, relational database structure designed for population surveillance. Each household is assigned a unique HHID and each individual an INDID, enabling linkage of demographic episodes, survey data, and clinical measurements to calculate person-time at risk and event rates. Field tablets store encrypted records locally during the day; at the end of each field shift, data are synchronized via secure, password-protected connections to a central cloud-based server hosted on institutional infrastructure. Built-in validation rules (range checks, mandatory fields, and cross-variable consistency checks) run at the point of entry, with additional automated scripts on the server to detect duplicates, outliers, and missingness. Data collectors receive intensive training on the electronic tools, household listing procedures, eligibility screening, physical measurements, point-of-care devices, ethics, and field record keeping, and they are supervised by field coordinators who conduct random spot checks and reinterviews for quality control. All equipment is calibrated regularly according to manufacturer recommendations, and standard operating procedures govern every step from mapping and enrollment to follow-up and data cleaning.

To maintain secure linkage between participant identity and the assigned INDID, a separate access-restricted identity register mapping personal identifiers (full name, date of birth, and sex) to the INDID is maintained on an encrypted, password-protected server, accessible only to the principal investigator and senior data manager. All analytical datasets contain INDIDs only, with no direct personal identifiers, ensuring participant confidentiality. At each follow-up visit, fieldworkers verify the participant’s name, date of birth, and household ID verbally before accessing their longitudinal record, confirming correct identity linkage.

### Data Analysis

The analytic plan encompasses descriptive statistics, robust prevalence and incidence estimations, and comparative analyses designed to quantify rural versus urban differences. Statistical software, primarily Stata (version 18; StataCorp LLC), will be employed. Numeric variables will be summarized using medians and IQRs, while categorical variables will be summarized using proportions. Logistic regression analyses (univariable and multivariable) will be used to identify and assess risk factors associated with NCDs, employing a hierarchical approach and stepwise backward elimination. The calculation of birth, mortality, prevalence, and incidence rates will be executed accordingly, and chi-square tests for trend will be used to analyze changes in NCD burden over time. Advanced statistical techniques, including regression and survival analyses, will facilitate robust estimation of NCD determinants and epidemiological transitions between the distinct study settings.

### Ethical Considerations

Ethical approval for this longitudinal surveillance system has been formally secured from the Medical Research Council of Zimbabwe (MRCZ/A/3191). The protocol strictly mandates that informed written consent is obtained from all participants prior to enrollment, ensuring that they fully comprehend the voluntary nature of their participation and their absolute right to withdraw without consequence. Confidentiality and privacy are strictly maintained through the use of unique participant identification codes and secure, encrypted data storage systems, ensuring no disclosure of personal details to third parties without express consent, unless legally compelled. Specialized consent procedures are in place for the collection and storage of blood and urine samples (both temporary and indefinite storage for future studies), as well as for the use of photographs, audio recordings, or video recordings. Participants diagnosed with clinically significant conditions (eg, hypertension or diabetes) are promptly referred to the nearest health facilities via prespecified referral protocols. Verbal autopsies follow international norms, requiring proxy consent from the next of kin.

### Dissemination

The study findings will be disseminated through a multifaceted approach targeting diverse audiences. Results will be communicated to the scientific community via peer-reviewed publications and presentations at national and international academic conferences and workshops. Crucially, condensed policy briefs will be generated and distributed to national health authorities and policymakers to directly inform policy decisions. Community-level engagement will include feedback sessions, utilization of local media (eg, radio programs), and social media platforms to ensure accessibility of findings to the public. All study tools, standard operating procedures, and supplementary information will be included as annexes to maximize research transparency and reproducibility.

## Results

The Zimbabwe HDSS was granted ethical approval by the Medical Research Council of Zimbabwe on July 30, 2024. Community profiling and household mapping were completed in both sites in the third quarter of 2024. Baseline data collection was completed in Mt Darwin (rural site) in December 2024 and in Bindura (urban site) in March 2025. Data cleaning and quality assurance procedures for the baseline dataset were initiated in April 2025, and statistical analysis of baseline prevalence, risk factor distributions, and rural-urban comparisons is projected for completion by the first quarter of 2026, with findings expected to be submitted for publication by the end of 2026. The first annual longitudinal follow-up round is planned for the second quarter of 2027, subject to funding availability. Subsequent follow-up rounds are scheduled annually through 2029, with longitudinal incidence estimates, demographic event rates, and NCD trend analyses expected to be published progressively from 2026 onward.

## Discussion

### Anticipated Contributions and Impact

The establishment of a comprehensive HDSS encompassing both rural and urban locales in Mashonaland Central Province is poised to yield findings with transformative potential for public health policy, program design, and research [32,33]. This system’s fundamental contribution lies in its capacity to generate high-quality, longitudinal data necessary for the monitoring of NCDs, migration, and demographic events—a critical gap currently present in Zimbabwe’s routine health information infrastructure [34]. Unlike cross-sectional surveys, the systematic, repeated population-based data collection permits the detection of subtle, emerging health trends and the robust monitoring of persistent socioeconomic inequalities over time [10,35].

A defining methodological asset is the dual-site, rural-urban design (Mt Darwin and Bindura). This design facilitates direct, empirical comparisons, providing nuanced evidence on how geographical context, migration patterns (including circular migration), and social determinants interact with NCD risk and disease progression [36,37]. For instance, the platform can contrast rural challenges—such as restricted access to medical care and delayed diagnosis—against urban epidemiological profiles, which often exhibit faster lifestyle transitions, increased environmental risk exposures, and elevated rates of obesity or metabolic syndrome [36]. The inclusion of dedicated modules covering behavioral risk factors, health care utilization, and cause-specific mortality via verbal autopsy ensures that emerging NCD patterns are thoroughly contextualized [38,39]. The digital platforms employed, featuring real-time data validation and immediate uploads, ensure both timeliness and the capability to respond rapidly to local public health priorities [40,41].

The intrinsic flexibility of the HDSS model provides a robust infrastructure for launching nested substudies and facilitating specialized research modules [10]. This adaptability allows for targeted investigation into specific NCD phenotypes or vulnerable age cohorts and supports the future establishment of a biobank for advanced biomarker and genetic studies, all anchored within the longitudinal cohort framework [37]. Critically, the potential for linking real-world surveillance data with facility-level health records addresses a current research challenge, enabling longitudinal evaluation of care quality, patient trajectories, and overall health system performance—outcomes typically inaccessible through standard administrative datasets alone [42,43].

A substantial anticipated contribution involves focusing research attention on the social and economic determinants of NCD outcomes, particularly how these factors differ and intersect across the rural-urban continuum [37]. By tracking variables such as educational attainment, occupational status, intrahousehold conditions, and individual and household migration, the HDSS will generate pivotal insights into identifying the most vulnerable demographic groups, pinpointing areas of unmet health need, and understanding how environmental conditions (eg, water and sanitation) and socioeconomic disadvantages influence NCD risk [44,45]. This evidence is paramount for guiding equitable, sustainable public health responses in rapidly urbanizing districts where new health inequities emerge due to constant population mobility [46].

The success of this HDSS is intrinsically tied to its ongoing engagement with local communities, health authorities, and key stakeholders in both rural and urban settings [47]. The methodological reliance on participatory approaches ensures that surveillance findings are communicated in accessible formats and, importantly, that community input is actively integrated to shape subsequent research priorities and data collection efforts [47]. Structured community engagement mechanisms, including site-specific Community Advisory Boards, postround community feedback meetings, and preround formative assessments, ensure that community input actively shapes data collection methods, dissemination formats, and research priorities. These participatory mechanisms are distinct from the formal institutional collaborations (described below) with academic, government, and health system partners, which focus on scientific, technical, and policy objectives. This creation of continuous feedback loops enhances the practical relevance, acceptability, and credibility of the evidence, fostering a sense of shared responsibility necessary for translating surveillance findings into local policy and practice [48,49]. Ultimately, by fostering strong collaborative ties across academic institutions, government bodies (including the Ministry of Health and Child Care), and health care providers, the HDSS aims to serve as a regional model for policy-responsive, sustainable, and ethically sound surveillance, building the local capacity necessary to accelerate Zimbabwe’s path toward universal health coverage and the Sustainable Development Goals [50,51].

### Strengths and Limitations

The principal strengths of this rural-urban HDSS lie in its inherent methodological design, which provides longitudinal, context-rich insights into the dynamics of NCDs across contrasting environments. The systematic, repeated tracking of vital events, disease trends, and risk factors, employing harmonized, standardized methods in both rural and urban settings, facilitates robust comparisons of health transitions and allows for the differential assessment of intervention impacts—capabilities that fundamentally distinguish it from conventional cross-sectional designs. Comprehensive enumeration, coupled with proactive community engagement within distinct environments, alongside the application of validated digital data collection tools (REDCap), ensures the acquisition of high-quality, relevant longitudinal information. This dual-site structure is particularly valuable for identifying emerging health disparities, enabling the tailoring of evidence-based public health responses specific to each context and significantly enhancing the system’s contribution to multisite analytic collaborations and sustainable research capacity development in Zimbabwe.

Nevertheless, this intensive methodological approach presents several limitations that demand vigilant management. The purposive selection of surveillance sites, designed to capture key rural and urban contrasts, means that the findings may not achieve full generalizability to Zimbabwe’s highly heterogeneous national population or to other heterogeneous regions. Ongoing operational challenges include managing participant attrition, particularly loss to follow-up, which may be exacerbated in mobile urban or periurban populations, and navigating the complexities inherent in conducting intensive longitudinal surveys across diverse community settings. The current protocol captures mobile phone ownership and network availability as basic proxy indicators of digital access; however, it does not comprehensively assess digital readiness, including smartphone penetration, broadband affordability, charging infrastructure, or internet literacy. Given the rapidly evolving digital landscape in Zimbabwe, including the emergence of satellite-based internet services and the growing use of connected devices for chronic disease self-management, a more comprehensive digital readiness module is planned for incorporation in future surveillance rounds. Furthermore, the considerable resource demands necessary for sustaining the dual-site system, alongside potential risks related to self-report bias concerning sensitive health behaviors and the necessity of maintaining stringent data security protocols across disparate environments, represent significant constraints. Self-report bias for sensitive health behaviors (including sexual behavior, alcohol consumption, and mental health) remains a methodological constraint of interviewer-administered surveys. Evidence from randomized trials demonstrates that Audio Computer-Assisted Self-Interviewing (ACASI) significantly reduces social desirability bias for sensitive topics in longitudinal research [52,53]. Our study will offer literate participants the option to read selected sensitive questions on the tablet and enter their responses directly rather than responding orally to the interviewer. This partial self-administered approach is intended to reduce response bias for sensitive topics, although it is not a full ACASI implementation. Resources permitting, we plan to expand the use of self-administered or ACASI-style modules in future rounds to further reduce self-report bias. Through careful methodological stewardship and adherence to established data quality management procedures, the HDSS is positioned to mitigate these limitations, providing a robust platform for context-sensitive evidence generation.

### Collaboration

The project maintains a specific emphasis on fostering collaborations, both multi-institutional and interdisciplinary, that aim to advance scientific knowledge concerning context-specific NCD patterns, intervention effectiveness, and rural-urban differentials. For the continued success and sustainability of the HDSS, additional collaboration and funding are both welcomed and essential. The baseline phase has been funded by the Fogarty International Center and the National Institute of Dental and Craniofacial Research of the National Institutes of Health (award D43 TW011968) through the ENRICH (Enhancing Non-Communicable Diseases Research and Innovation Capacity in Harare, Zimbabwe) program, in partnership with the University of Zimbabwe and the Ministry of Health and Child Care. Additional partners and donors are welcomed to support the continuation and scaling of follow-up phases. This support will play a key role in enabling the rigorous implementation and scaling of follow-up phases critical to achieving long-term research and public health objectives.

### Future Plans

Future plans for the HDSS include the continuation of biannual longitudinal follow-up rounds over a 5-year period to monitor the incidence and prevalence of common NCDs and update data on key risk factors and social determinants. Efforts will focus on analyzing trends in births, deaths, migration, and health-related behaviors to understand shifts in NCD patterns within rural and urban contexts. The long-term strategic trajectory of the HDSS includes plans to incrementally expand the geographic coverage to incorporate additional rural and urban wards. This expansion aims to enhance the system’s capacity to capture broader regional variations and longitudinal shifts in NCD burden.

Mt Darwin (rural) and Bindura (urban) were purposively selected as typical districts within Mashonaland Central Province, reflecting common rural agricultural and urban mixed-economy contexts and existing collaborations with the Ministry of Health and Child Care and the University of Zimbabwe. This initial phase is therefore designed to establish a feasible dual-site HDSS that captures the primary contrast between rural and urban settings, rather than to be statistically representative of all rural or all urban districts nationally. To address this limitation, future phases of the HDSS are planned to extend surveillance to additional rural and urban districts in Zimbabwe, thereby widening geographic coverage and enabling assessment of variation within rural areas and within urban areas, as well as between them.

Future activities will also focus on integrating new, specialized modules dedicated to capturing data on environmental exposures, performing comprehensive mental health assessments, and facilitating more detailed NCD phenotyping. The HDSS aims to expand geographic coverage to include additional districts for broader regional representation. New data collection modules will be incorporated to capture environmental exposures, detailed mental health assessments, and refined NCD phenotyping. These activities will enable robust rural-urban comparisons and provide evidence to guide targeted public health policies and interventions, particularly for vulnerable populations.

### Conclusion

The establishment of this HDSS fills a significant evidence gap in Zimbabwe’s national health information infrastructure and represents a critical advancement in public health research, particularly for NCDs. This longitudinal, population-based cohort is designed to continuously monitor the prevalence, incidence, and risk factors of NCDs over a 5-year period (2024‐2029) across both rural (Mt Darwin) and urban (Bindura) communities, providing a robust dual-site methodology for empirically comparing disease epidemiology and determinants—such as migration, socioeconomic status, and environmental exposures. Through the systematic implementation of standardized and repeated collection of physical and biochemical measurements, the HDSS generates high-quality, actionable longitudinal data that are essential for discerning complex NCD patterns, identifying emerging trends, and addressing underserved populations. Ultimately, the knowledge generated from this surveillance effort will inform targeted, equitable intervention strategies, support evidence-based health system planning, and accelerate progress toward national and regional NCD control and prevention goals.

## Abbreviations

ACASI: Audio Computer-Assisted Self-Interviewing
BP: blood pressure
DHS: Demographic and Health Survey
EAs: Enumeration Areas
ENRICH: Enhancing Non-Communicable Diseases Research and Innovation Capacity in Harare, Zimbabwe
GIS: geographic information system
HDSS: Health and Demographic Surveillance System
HHID: household identification
INDID: individual identification
NCD: noncommunicable disease
REDCap: Research Electronic Data Capture
WHO: World Health Organization
WHO STEPS: WHO STEPwise approach to NCD risk factor surveillance
